# Influence of Oilseed Rape Seed Treatment with Imidacloprid on Survival, Feeding Behavior, and Detoxifying Enzymes of Mustard Aphid, *Lipaphis erysimi*

**DOI:** 10.3390/insects10050144

**Published:** 2019-05-20

**Authors:** Fang Huang, Zhongping Hao, Fengming Yan

**Affiliations:** 1 Huzhou Custom, Huzhou 313000, China; 2 Anhui Province Key Laboratory of Crop Quality Improvement, Crop Research Institute, Anhui Academy of Agricultural Sciences, Hefei 230031, China; hzp5187@sina.com; 3 College of Plant Protection, Henan Agricultural University, Zhengzhou 450002, China; fmyan@henau.edu.cn

**Keywords:** *Lipaphis erysimi*, oilseed rape, imidacloprid, coating agent, feeding behavior

## Abstract

Imidacloprid application, as a seed coating agent on oilseed rape, is recommended to control mustard aphid, *Lipaphis erysimi* (Kaltenbach) (Hemiptera: Aphididae). In this study, responses of *L. erysimi* were investigated, including survival, feeding behavior, and detoxifying enzymes, on the oilseed rape seedlings grown from seeds coated with imidacloprid at rates of 6, 12, or 18 g active ingredient (a.i.)/kg seed. The results showed that the aphids’ survival rate, together with that of the progeny of the survivors, on the seed-treated seedlings significantly decreased. This indicates that the aphid population in fields can be suppressed effectively. The electrical penetration graph (EPG) technique was used to record aphid feeding behaviors on two-, four-, and six-leaf stages of oilseed rape seedlings that had been seed-coated with imidacloprid, and individual responses were revealed during the aphid feeding behavior. On the plants at the two-leaf stage, aphid feeding behaviors were influenced, showing decreased frequency of stylet penetration into the leaf (probe) or into the mesophyll cells (potential drops, pds for short), and shortened duration of stylet event in the leaf (probe) or in the phloem. On the plants at the four- and six-leaf stages, these impacts of imidacloprid were weakened; however, the saliva secretion duration in phloem was shortened to less than 5 min in all imidacloprid treatments. The activity of mixed-function oxidase in aphids maintained on the treated seedlings with imidacloprid was elevated. In conclusion, imidacloprid could be used as a seed coating agent for aphid control, but chemical resistance in aphids should not be ignored.

## 1. Introduction

Imidacloprid, a broad spectrum systemic chloronicotinyl insecticide, is effective against a wide range of piercing—sucking insect pests as an insect nerve antagonist. It has been registered for use in more than 120 countries as one of the most effective insecticides for aphids, leafhoppers, planthoppers, whiteflies, certain coleopterans, and micro-lepidopterans. Importantly, it has low mammalian toxicity [1]. Due to its enormous agricultural and economic importance, imidacloprid has been recommended and marketed worldwide for use in over 140 agricultural crops [1] and in forestry [2].

Aphids, as one of the most important targets of neonicotinoids, are effectively controlled by imidacloprid via spraying, drip-chemigation, or root-irrigation [3,4,5]. Even low systemic concentrations of imidacloprid in cabbage leaves have an antifeeding effect on aphids [6]. However, residues of imidacloprid may cause environmental pollution and threaten pollinator population [7]. Thus, imidacloprid is often recommended as a coating agent in seed treatments [8,9]. Effective control of aphids by imidacloprid seed-coating has been demonstrated in different pest-plant combinations. Wheat seedlings from seeds coated with imidacloprid had significant stomach toxicity and an antifeedant activity against wheat aphid (*Macrosiphum avenae*) [10,11]. Reduced density of *Rhopalosiphum padi* on maize seedlings [12], effective bio-efficacy of cotton seedlings against *Aphis gossypii* [13], increased mortality of cabbage aphid *Brevicoryne brassicae* on cabbage [14], and increased mortality of *Rhopalosiphum padi* on oat [15] were observed. However, some studies did not report similar observations. Seagraves and Lundgren conducted experiments and determined that the bioactivity of seed treatments against soybean aphids disappeared within 46 days after planting, prior to aphid populations damaging the crop [16]. Pons and Albajes described a population of *Metopolophium dirhodum* being enhanced on imidacloprid-coated maize after flowering [17]. Rapeseeds are usually coated by Gaucho^®^ (70% imidacloprid; Bayer Crop Science, Hangzhou, China) before sowing, but little data have been published about how imidacloprid seed-coating treatment influences aphids on oilseed rape seedlings.

In this study, we conducted experiments to reveal the effects of seed treatment with imidacloprid on aphids by investigating the aphids’ population development, monitoring aphids’ feeding behaviors, and detecting some enzymes that might reflect aphids’ resistance, in an attempt to provide basic information for effective control of mustard aphid on oilseed rape with imidacloprid seed-coating treatment.

## 2. Materials and Methods 

### 2.1. Insects and Plants

Insects and plants were used as described in our previous study [18]. Mustard aphids, *Lipaphis erysimi*, were collected from infested cabbage fields near Zhejiang Academy of Agricultural Sciences, Hangzhou, China, in July 2013, and were maintained as a laboratory colony under conditions of 25 °C, 75% relative humidity (R.H.), and a 16:8 h (light:dark; L:D) photoperiod. Xinyou-10, a cultivar of oilseed rape, was used as experimental material due to being cultivated over large areas in China.

### 2.2. Seed Treatments

Referring to our previous study [18], oilseed rape seeds were treated with imidacloprid (Gaucho 480FS, Gustafson LLC, Dallas, TX, USA) at rates of 6, 12, and 18 g active ingredient (a.i.) per kg seeds as treatment L (Low dose), M (Median dose), and H (High dose), respectively, and untreated seeds were set as controls (CN). Plants were grown in plastic pots (diameter 15 cm, depth 17 cm) filled with a mixture of sand/soil/peat/perlite (2:1:3:3) in a climate-controlled chamber at 25 ± 2 °C under 400 W high intensity discharge lamps with a photoperiod of 16:8 h (L:D). At 2-, 4- and 6-leaf stages, the seedlings were used for experiments.

#### 2.2.1. Aphid Survival

The two-leaf seedlings (area of the third leaf was no more than 0.25 mm^2^) were used for aphid population investigation for L, M, H, and CN treatments. In each treatment, 30 newly emerged apterous adult aphids were carefully transferred onto a seedling with a fine hair paintbrush. To avoid aphid escape, the plant was caged in a porous (80 mesh) transparent plastic cup (ca. 40 cm height, and ca. 20 cm in diameter; Zhejiang Hongda Plastic Co., Ltd., Hangzhou, China). In each treatment, 10 plants were used. These plants were kept under the same conditions as mentioned above. After 1 week, the number of dead and live tested aphid individuals were recorded, together with counts of their offspring (including live and dead nymphs) on each plant. Treatments of each rate of imidacloprid (including untreated set) were replicated four times. The experiment was continued until plants reached the 6-leaf stage.

#### 2.2.2. Monitoring of Aphid Feeding Behavior

Electrical penetration graphs (EPGs) are useful for monitoring aphid feeding behaviors on plants, and EPG output (waveforms and variables) can reflect plant resistance to the aphids [19]. Surviving aphids from experiment 2.2.1 were carefully transferred onto aphid-free seedlings of the corresponding developmental stages in each treatment (L, M, H, and CN) maintained in the climate-controlled chamber. After 12 h, the aphids’ feeding behaviors were monitored with EPG.

Stylet penetration activities (i.e., feeding behaviors) of aphids were recorded using a 4-channel direct-current electrical penetration graph (DC-EPG) system (Giga-4; EPG systems, Wageningen University, Wageningen, the Netherlands) with an A/D card (DI-710, Dataq Instruments Inc, Akron, OH, USA), and analyzed following the methods and the waveform recognition standards developed by Tjallingii [19]. An aphid was gently attached to an insect electrode, a gold wire (12.5 μm diameter, 2 cm long) with silver glue, and placed onto a lower surface of a plant leaf. Recordings were conducted inside a Faraday cage in the laboratory at 27 ± 2 °C. The recordings were started around 8:30 and continued for 8 h under florescent light (ca. 1000 lux). A separate aphid and a separate plant were used for each recording.

From each treatment, 20 successful recordings were selected for characterization and analysis of the EPG waveforms using the EPG analysis worksheet [20]. Aphids are phloem-feeding insects [21], thus data analysis of the aphids’ feeding behaviors, therefore, focused on total stylet-probing time, pds in the mesophyll cells, and phloem-associated activities.

#### 2.2.3. Detoxifying Enzymes

Based on the results of the above experiments, the aphids in treatments L or M, which succeeded in developing colonies and survived to the time when the seedlings had 6-leaves, were used for detection of enzymatic activities. The aphids reared on the untreated seedlings were set as controls.

Activities of 3 major detoxifying enzymes—carboxylesterase, glutathione-*s*-transferase, and mixed-function oxidase—were tested. In each detection, 10 adult aphids frozen in liquid nitrogen were homogenized in 1 mL of 0.1 M phosphate buffer (pH 7.0) at 4 °C, then the homogenate was centrifuged at 13,000× *g* for 15 min at 4 °C, and the supernatants were used to analyse enzyme activities. Detections for each enzyme were replicated 4 times.

Following Cui et al. [22] with some modifications, total carboxylesterase activities were quantified using a Beckman DU-800 spectrophotometer (Becton-Dickinson, Fullerton, CA, USA). Glutathione-*s*-transferase activities were determined following the method used by Sun et al. [23]. Enzyme activities of the mixed-function oxidase were measured using the method referred to in Sintim et al. [24] toward ρNA. Total protein concentrations in the above-mentioned supernatants were determined using Bradford’s method [25].

### 2.3. Statistics

Data were analyzed using SPSS 10 software (SPSS Ltd, Chicago, IL, USA). Proportion data associated with aphid mortality (except zero value of the CN treatment) were arcsine-transformed prior to ANOVA analysis (zero was included herein) followed by Tukey’s test [26], but the original percentage data were used for presentations. Values of aphid counts were analyzed in the same way.

EPG data were transformed where appropriate (square-root transformation for number of occurrences and natural log transformation for duration). Significant differences between data at the same stage of the treatment and the control were checked using the *t*-test.

## 3. Results

### 3.1. Effect of Imidacloprid Seed Treatments on L. erysimi Survival

**Figure 1:**
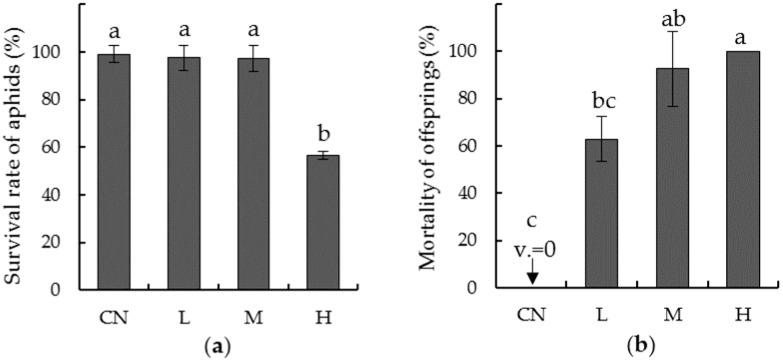
Effect of imidacloprid seed treatments on (**a**) aphids’ and (**b**) their offspring’s survival rate. CN: Control; L: Seed coated by low imidacloprid dose; M: Seed coated by medium imidacloprid; H: Seed coated by high imidacloprid dose. Bars indicate standard errors. Bars with the same letters are homogeneous groups or are not statistically different. v. = value.

Levels of imidacloprid a.i. had significant effects on the survival of *L. erysimi* on oilseed rape (Figure 1). For the tested aphid generation (Figure 1a), survival rates of aphids in treatments L and M were unaffected; whereas aphids in treatment H showed a significant decrease in survival rate to near 60% (F_3,39_ = 151.234, *p* < 0.05). For the next generation (Figure 1b), all three seed-coating treatments caused significantly higher mortality in nymphs compared to the zero mortality in the control (F_3,39_ = 66.771, *p* < 0.05), i.e., about 50–70% in treatment L, 80–100% in treatment M, and 100% in treatment H.

**Figure 2:**
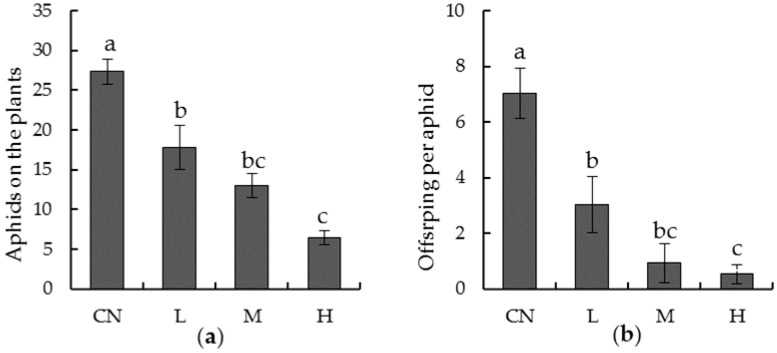
Survival of (**a**) tested aphids and (**b**) their progeny on oilseed rape seedlings that had been seed treated with imidacloprid. CN: Controls; L: Seed coated with low imidacloprid dose; M: Seed coated by medium imidacloprid dose; H: Seed coated by high imidacloprid dose. Bars indicate standard errors. Data were analyzed using analysis of variance (ANOVA) followed by Tukey’s test, and different lower-case letters indicate significant differences (*p* < 0.05).

To estimate the repellent effects, aphids feeding on the plants were counted (Figure 2). For the tested generation (Figure 2a), counts of aphids that continued feeding on the plants significantly decreased in the chemical treatments (F_3,159_ = 24.436, *p* < 0.05). As the treating doses increased, the remaining individuals decreased significantly from 27 (CN) to 18 (treatment L), 13 (treatment M), and 6 (treatment H). A similar occurrence was observed in the remaining aphids of the next generation: The average number of nymphs produced by the surviving tested aphids significantly decreased (F_3,159_ = 81.303, *p* < 0.05). Compared with the control, average offspring per tested aphids in L-treatment significantly decreased by 65.2%, 47.7% in M treatment, and 23.5% in H treatment, respectively (Figure 2b).

### 3.2. Effect of Imidacloprid Seed Treatments on L. erysimi Feeding Behavior

The above results show that the high chemical treatment dose could kill all the tested aphids, whereas about half the testing colonies remained alive with the low or median dose; there was no significant difference between the latter two treatments. Thus, only EPG data recorded from the M-treatment are represented here (Table 1). According to the comparisons between the treatments and the controls at the corresponding stages, *L. erysimi* had more probes but less total probing time on the treated seedlings at the two-leaf stage, which suggests a much shorter duration per probe in the imidacloprid treatment; however, no such phenomenon was observed at the four- or six-leaf stages.

In seed-coating treatments, the total time of aphids’ penetration in mesophyll cells (duration of pds) was unaffected in two-leaf seedlings, but significantly decreased by 50% and 20% on four- and six-leaf seedlings, respectively. Numbers of aphid stylet punctures into mesophyll cells (numbers of pd) were significantly reduced on the seed-treated seedlings. At the two- or four-leaf stages, the numbers of stylet puncture were nearly half that of the corresponding controls, whereas at the six-leaf stage, the punctures increased but were still significantly lower than the control.

Aphid feeding behaviors associated with sieve elements were also significantly affected (Table 2). Throughout the EPG monitoring, the salivation durations of aphids in the seed-treated seedlings were significantly inhibited. Salivation frequency of aphids on the seedlings at the two- or four-leaf stages did not significantly differ from the corresponding control, whereas that at the six-leaf stage was significantly lower. Total time of phloem feeding on the seed-treated seedlings significantly shortened up to no more than 1 min on the two-leaf seedlings, and to half that of the control on the four-leaf seedlings, but on the six-leaf seedlings, the data of the treatments were similar to the controls. The frequency of phloem feeding significantly decreased on the two-leaf seedlings; however, no significant differences on the four- or six-leaf seedlings were observed compared to the control.

### 3.3. Effect of Imidacloprid Seed Treatments on L. erysimi Detoxifying Enzymes

**Figure 3:**
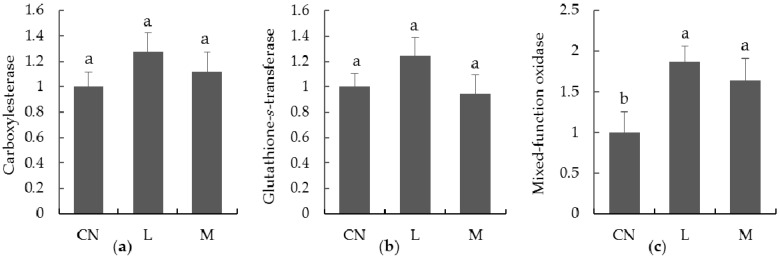
Effect of imidacloprid seed treatments on aphids’ detoxifying enzymes (average ± standard error), (**a**) carboxylesterase, (**b**) glutathione-*s*-transferase, and (**c**) mixed-function oxidase. CN: The controls; L: Seed coated by imidacloprid of low dose; M: Seed coated by imidacloprid of median dose. Different small letters show significant difference (*p* < 0.05).

Seed-coating treatments had some effects on aphids’ detoxifying enzymes (Figure 3). For carboxylesterase and glutathione-*s*-transferase, no significant changes were observed in treatments compared to the control (carboxylesterase: F_2,29_ = 2.412, *p* > 0.05; glutathione-*s*-transferase: F_2,29_ = 3.228, *p* > 0.05); whereas for mixed-function oxidase, seed coating treatments with imidacloprid significantly elevated the enzyme activities with no significant differences (F_2,29_ = 16.502, *p* < 0.05) between dose treatments.

## 4. Discussion

On oilseed rape with imidacloprid seed-coating treatment at the normally recommended a.i. concentrations, the aphid survival test results showed that adult aphids’ mortalities hardly reached more than 50%, even at the highest concentration, but a significant repellent effect of seed-coating with imidacloprid was observed, even at the lowest concentration. This repellent effect was also observed in the feeding behavioral analysis of cotton aphid *Aphis gossypii* [27]. Although more than half of the tested aphids survived on the seed-treated seedlings, the maternal fecundity significantly decreased. This negative effect on aphid population establishment was also observed on imidacloprid-treated cotton, corn, cereal, sugar beet, and other crops [28].

The EPG data confirmed that seedlings that had received seed-coating treatments had a repellent effect on aphids, especially based on the observations on two-leaf seedlings: (1) As an overview of probes, decreased total time and increased frequency meant the average probe duration significantly shortened, representing a decreased feeding efficiency; (2) for ingestion attempts in mesophyll cells, more attempts indicated that the tested aphid spent more time seeking a suitable ingestion location; (3) the salivation secretion, which was proven to be the key process in virus-transmission by aphids [29], was also inhibited; and (4) the ingestion behavior was inhibited, although the significance was not detected at the four- or six-leaf stages. Previous EPG recordings revealed that, on artificial membranes containing imidacloprid at low concentrations, *Myzus persicae* probed more often before ingestion than aphids on control sachets [30]. These findings provide evidence that imidacloprid absorbed through roots could have a long-term systemic efficiency in decreasing aphid damage to plants by inhibiting the aphids’ feeding behaviors. Inhibition of aphids’ stylet activities in the plant tissues could hamper the transmission of viruses vectored by aphids, as seed treatment with Gaucho^®^ was found to reduce three aphid-borne viruses in legume crops [31]. Thus, transmission of viruses by aphids should be suppressed in imidacloprid seed-treatment plants of oilseed rape.

However, the phloem feeding variables in EPG-recorded feeding behaviors of aphids on the treated seedlings suggest some different indications. Total time of aphid phloem feeding significantly shortened on two-leaf treated seedlings, but then increased on four- and six-leaf seedlings. Phloem feeding is the most important variable or indicator to show how the aphids fit to their host plants [32,33]. Thus, increased phloem feeding time indicates that the aphids surviving on the four- or six-leaf seedlings might have recovered their feeding activity even up to a similar level as the controls, which could result from resistance development in aphids [34].

Pest resistance monitoring is important in the pesticide application strategy. Given biological characteristics, aphids are predestined to be living factories that inactivate insecticides [35]. Resistance development is based on genetic changes in the pest population, leading to many physiological changes, one of which is an elevation of detoxifying enzyme activities [35]. Mixed-function oxidase was found to be the most important enzyme conferring imidacloprid resistance of the aphid *M. persicae* [36], whereas glutathione-*s*-transferase [37] or carboxylesterase [38] did not change in the imidacloprid resistant strains of *A. gossypii*. In our present study, only mixed-function oxidase, not glutathione-*s*-transferase or carboxylesterase, was significantly elevated, which indicates that *L. erysimi* herein had developed resistance to imidacloprid to some extent. However, this inference needs to be confirmed in further field and laboratory investigations.

## 5. Conclusions

Oilseed rape seedlings produced from seeds that were seed-coat treated with imidacloprid provided effective aphid population suppression, evidenced by decreased survival and reduced fecundity in the survivors. EPG results showed that aphid feeding behaviors: (1) At the two-leaf stage were characterized by decreased frequency and duration of stylet penetration into the leaf (probing) as well as into the mesophyll cells (pds) or the phloem; and (2) at the four- and six-leaf stages were characterized by shortened duration of saliva secretion into the phloem. Aphids maintained on seedlings of imidacloprid-treated seeds demonstrated elevated levels mixed-function oxidases. In conclusion, imidacloprid used as a seed coating agent could provide good control of aphids, but the appearance of pesticide resistance should not be ignored.

## Figures and Tables

**Table 1:** Effect of imidacloprid seed treatments on aphid feeding behaviors in overview of probing.

EPG Variable	Stage	Mean ± SE ^1^		*p*-Value
		Treated	Untreated	
Number of probes	Two-leaf	52.93 ± 13.47	17.18 ± 3.37	<0.05
	Four-leaf	17.16 ± 2.89	15.45 ± 1.68	>0.05
	Six-leaf	17.52 ± 2.57	17.76 ± 1.98	>0.05
Duration of probe (h)	Two-leaf	4.07 ± 0.49	5.52 ± 0.08	<0.05
	Four-leaf	5.70 ± 0.05	5.53 ± 0.10	>0.05
	Six-leaf	5.74 ± 0.03	5.70 ± 0.03	>0.05
Duration of pds (s)	Two-leaf	539.23 ± 69.86	608.80 ± 83.26	>0.05
	Four-leaf	360.77 ± 60.22	667.43 ± 57.60	>0.05
	Six-leaf	609.87 ± 55.07	768.56 ± 68.28	>0.05
Number of pds	Two-leaf	86.14 ± 19.33	155.00 ± 23.34	<0.05
	Four-leaf	71.83 ± 11.43	166.36 ± 14.96	<0.05
	Six-leaf	133.87 ± 12.88	162.35 ± 14.93	<0.05

^1^ SE = standard error.

**Table 2:** Effect of imidacloprid seed treatments on aphids’ feeding behaviors in phloem-associated elements.

EPG Variable	Stages	Mean ± SE		*p*-Value
		Treated	Untreated	
Time of saliva secretion in phloem (min)	Two-leaf	1.27 ± 0.68	18.76 ± 3.00	<0.01
	Four-leaf	1.37 ± 0.87	18.64 ± 2.68	<0.01
	Six-leaf	1.35 ± 0.52	18.22 ± 1.08	<0.01
Number of saliva secretion in phloem	Two-leaf	1.24 ± 0.34	1.92 ± 0.25	>0.05
	Four-leaf	4.00 ± 0.47	4.80 ± 1.20	>0.05
	Six-leaf	5.09 ± 0.41	9.50 ± 1.21	<0.01
Time of sap ingestion in phloem (min)	Two-leaf	0.15 ± 0.04	3.59 ± 9.16	<0.05
	Four-leaf	34.23 ± 4.02	65.80 ± 15.52	>0.05
	Six-leaf	107.75 ± 20.94	108.98 ± 22.98	>0.05
Number of sap ingestion in phloem	Two-leaf	0.25 ± 0.16	1.35 ± 0.34	<0.05
	Four-leaf	3.13 ± 0.81	3.54 ± 0.89	>0.05
	Six-leaf	3.61 ± 0.93	4.5 ± 0.16	>0.05
